# Renal cyst growth is attenuated by a combination treatment of tolvaptan and pioglitazone, while pioglitazone treatment alone is not effective

**DOI:** 10.1038/s41598-020-58382-z

**Published:** 2020-02-03

**Authors:** Anish A. Kanhai, Hester Bange, Lotte Verburg, Kyra L. Dijkstra, Leo S. Price, Dorien J. M. Peters, Wouter N. Leonhard

**Affiliations:** 1 Department of Human Genetics, Leiden University Medical Center, Leiden, the Netherlands; 2 OcellO B.V, Leiden, the Netherlands; 3 Department of Pathology, Leiden University Medical Center, Leiden, the Netherlands

**Keywords:** Nephrology, Polycystic kidney disease, Polycystic kidney disease

## Abstract

Autosomal Dominant Polycystic Kidney Disease (ADPKD) is one of the most common monogenic disorders, characterized by the progressive formation of fluid-filled cysts. Tolvaptan is an approved drug for ADPKD patients, but is also associated with multiple side effects. The peroxisome proliferator-activator receptor gamma (PPARγ) agonist pioglitazone slows disease progression in the PCK rat model for PKD. Here, we tested whether a combination treatment of relevant doses of tolvaptan and pioglitazone leads to improved efficacy in an adult-onset PKD mouse model. Tolvaptan indeed slowed PKD progression, but the combination treatment was not more effective than tolvaptan alone. In addition, although pioglitazone raised plasma levels of its surrogate drug marker adiponectin, the drug unexpectedly failed to slow PKD progression. The pioglitazone target PPARγ was expressed at surprisingly low levels in mouse, rat and human kidneys. Other pioglitazone targets were more abundantly expressed, but this pattern was comparable across various species. The data suggest that several potential pharmacokinetic and pharmacodynamic (PK/PD) differences between different species may underlie whether or not pioglitazone is able to slow PKD progression. The ongoing phase II clinical trial with low-dose pioglitazone treatment (NCT02697617) will show whether pioglitazone is a suitable drug candidate for ADPKD treatment.

## Introduction

Autosomal Dominant Polycystic Kidney Disease (ADPKD) is one of the most common monogenic disorders, affecting 1 in 2500 individuals^1^. The most prominent disease feature is the progressive formation of fluid-filled cysts, mainly in the kidneys and less frequent in liver and pancreas^2^. In the majority of patients, the cause of disease is a mutation in the *PKD1* (±85%) or *PKD2* (±15%) gene, encoding for either polycystin-1 or polycystin-2^3,4^. Although the exact mechanisms are not yet completely understood, both *PKD1* and *PKD2* mutations have been shown to dysregulate many intracellular signalling pathways including mammalian target of rapamycin (mTOR), 5’ adenosine monophosphate-activated protein kinase (AMPK), transforming growth factor-beta (TGF-β) and extracellular signal-related kinase (ERK)^5–12^. Also, altered fluid secretion involving the cystic fibrosis transmembrane conductance regulator (CFTR) and metabolic alterations (increased glycolysis and reduced fatty acid oxidation) have been reported to contribute to disease progression^13–16^. Over the recent years, various interventions based on correcting dysregulated intracellular signalling have been tested in clinical trials^17–21^. So far, only the vasopressin V2 receptor antagonist tolvaptan (Jinarc^®^), which lowers intracellular cyclic AMP (cAMP) levels in the collecting duct segment of the kidney, was convincingly shown to slow disease progression in patients and is currently available in multiple countries as a treatment option^22–24^.

While tolvaptan is effective in delaying the loss of renal function, the drug is accompanied by side effects, including polyuria and liver toxicity^22–24^, restricting the use of the drug to a subset of ADPKD patients. Since ADPKD is a disease with a progressive nature, patients might require life-long treatment, which could start at an early stage of the disease. At this stage, kidney function and overall physical health are not yet significantly compromised. Therefore, it is vital, as well as challenging, to develop interventions that are both effective and safe. One option for improving efficacy and safety, is to use a combination of drugs to target the complex ADPKD signalling network from various angles simultaneously. In addition, potential synergism between the drugs may provide clinicians the opportunity to reduce the prescribed drug doses, thereby reducing unwanted side effects.

An interesting drug to consider for this strategy is the peroxisome proliferator-activator receptor γ (PPARγ) agonist pioglitazone. Originally used for treatment of type 2 diabetes, this drug, and other members of the thiazolidinedione (TZD) class, increase insulin sensitivity and alter both fatty acid and glucose metabolism^25^. The insulin-sensitizing effects of TZDs via PPARγ agonism are partly mediated by an increased expression of adiponectin, a hormone secreted by adipose tissue, which is known to regulate glucose levels^26,27^. This has been demonstrated in animal models and in human subjects^28–30^. Clinically used doses of pioglitazone have been reported to induce an approximate 2-fold increase of plasma adiponectin levels in healthy individuals and type 2 diabetes patients^31,32^. Through PPARγ, TZDs also regulate cell proliferation via ERK signalling, as well as fibrosis and inflammation through reduction of TGF-β levels in various renal disease models^33,34^. Moreover, pioglitazone also reduces CFTR gene expression in *in vitro* models of the principal cell^35^. As these processes and targets are also involved in ADPKD pathogenesis, pioglitazone and other TZDs have been tested in animal models for polycystic kidney disease (PKD). Maternal administration of high-dose pioglitazone (80 mg/kg/day) ameliorated the cystic phenotype of *Pkd1*^−/−^ mouse embryos and improved their survival^36^. However, pioglitazone treatment with 30 mg/kg food (∼ equalling 4 mg/kg/day) did not show any effect on renal function and cyst formation, but did increase survival in neonatal principal cell *Pkd1* knock-out mice^37^. Inhibition of PKD disease progression by TZDs has also been shown in the PCK rat model^38–40^. Based on these results, a phase II clinical trial has been initiated to explore whether low-dose pioglitazone treatment could slow ADPKD progression in patients (ClinicalTrials.gov number, NCT02697617).

Since tolvaptan is already an approved treatment for ADPKD patients and pioglitazone is currently tested in a clinical trial, we selected these drugs for a preclinical study to find out whether a combination treatment would result in an enhanced therapeutic efficacy in an adult-onset PKD mouse model (iKspCre-*Pkd1*^del^), compared to single-drug treatment with tolvaptan or pioglitazone. Our data show that tolvaptan and the combination treatment are equally effective in improving renal survival and slowing PKD progression. However, treatment with a clinically relevant dose of pioglitazone did not show any beneficial effect on PKD progression.

## Results

### Pioglitazone dose-dependently inhibits cyst swelling *in vitro*

**Figure 1:**
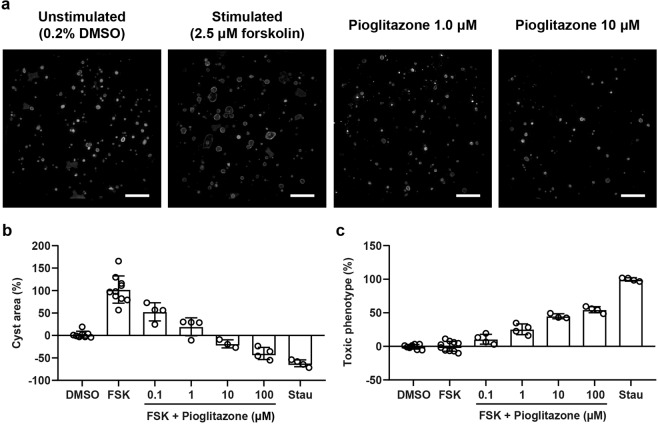
Pioglitazone dose-dependently inhibits cyst swelling in a non-toxic manner. **(a)** Representative images of 3D-cultured mIMCD3-*Pkd1*^−/−^ cysts (cytoskeleton, F-actin) exposed with vehicle control (unstimulated, 0.2% DMSO), forskolin (stimulated, 2.5 µM) or forskolin (2.5 µM) plus increasing concentrations of pioglitazone. These max z-projections of a complete well were taken with the ImageXpress Micro XLS imager and are shown for presentation purposes. **(b)** With co-exposure to forskolin, pioglitazone dose-dependently reduces cyst swelling in mIMCD3-*Pkd1*^−/−^ cysts. At the highest concentrations (10 and 100 µM), cyst size is lower than unstimulated vehicle control (0.2% DMSO). Cyst size is normalised based on unstimulated vehicle control (0.2% DMSO, set at 0%) and stimulated control (2.5 µM forskolin, set at 100%). **(c)** Multiparametric phenotypic measurements indicate that compared to the toxic control staurosporine, pioglitazone does not induce inhibition of cyst swelling through cytotoxicity up to 1 μM. Toxic phenotype is normalised based on unstimulated control (0.2% DMSO, set at 0%) and toxic control (0.25 μM staurosporine, set at 100%). Each dot represents a replicate per condition (n = 4). FSK: forskolin, Stau: staurosporine.

Previous research has shown that pioglitazone is effective in slowing disease progression in the PCK rat model for PKD and low-dose pioglitazone treatment is currently being tested in a clinical trial for ADPKD patients (ClinicalTrials.gov number, NCT02697617). To confirm the cyst reducing potential of pioglitazone, the drug was tested in an *in vitro* 3D cyst assay. Mouse inner medullary collecting duct *Pkd1*^−/−^ cells (mIMCD3-*Pkd1*^−/−^) were cultured in an extracellular matrix-based hydrogel in 384-well plates, and cyst swelling was induced by addition of forskolin, an inducer of cAMP production mediated via adenylyl cyclase. Cells were co-exposed to forskolin (2.5 µM) and pioglitazone at increasing concentrations (0.1–100 μM). In separate wells, staurosporine (0.25 μM) was used as a toxicity control compound, to evaluate whether reduction in cyst swelling by pioglitazone was not caused by cytotoxicity. Particularly to assess usefulness of compounds for ADPKD patients, it is important to make this distinction, because long-term treatment with cytotoxic drugs, and their associated side effects, are unlikely to be a viable therapy for ADPKD patients. Automated calculation of various phenotypic characteristics^41^ related to cyst area and cytotoxicity confirmed that pioglitazone dose-dependently reduces cyst swelling *in vitro* in a non-toxic manner up to 1 µM (Fig. 1a–c).

### Surrogate drug markers to determine relevant tolvaptan and pioglitazone doses for preclinical testing

**Figure 2:**
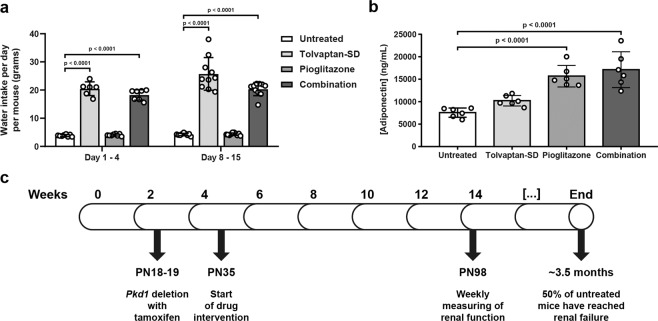
Tolvaptan and pioglitazone elevate surrogate drug markers *in vivo*. **(a)** Water intake per day per mouse (grams) in untreated and treated mice. Water intake was calculated by dividing the difference in water bottle weight by the number of mice per cage (n = 2 or 3) and the number of days between measurements. **(b)** Plasma adiponectin concentrations (ng/mL) in untreated and treated mice. Each data point represents the average plasma concentration of 1 mouse, measured in duplicate. Mice from each group were selected around the median of the 2KW/BW% ratios. **(c)** Graphical representation of the preclinical study. Tamoxifen (150 mg/kg) was administered to mice on PN18 & 19 to delete *Pkd1*. Treatments started at 5 weeks of age (PN35) and renal function was monitored from 14 weeks of age (PN98). The experiment was ended at the 50% ESRD time point (around 3.5 months of age). Data represent the mean ± SD. Significance was measured by one-way ANOVA followed by Tukey’s multiple comparisons test. PN: post-natal.

For our preclinical study, we first established drug doses for tolvaptan and pioglitazone. Tolvaptan spray-dried powder (tolvaptan-SD), a tolvaptan formulation designed to improve oral bioavailability, was administered at 0.15% in the diet, equalling the commonly used dose in preclinical research of 0.1% tolvaptan. Tolvaptan administration is known to be accompanied by massive aquaresis^42^. Because the aquaretic effect of tolvaptan is well known, we preferred water intake over urine output as a surrogate marker, because the latter requires housing in metabolic cages, which generally is perceived as rather stressful by the mice and could be a potential source for experimental variation. As expected, after the start of treatment, water intake increased substantially in the groups receiving tolvaptan-SD, confirming its efficacy (Fig. 2a). Clinically used doses of pioglitazone have been reported to induce an approximate 2-fold increase of plasma adiponectin levels in healthy individuals and type 2 diabetes patients^31,32^. We therefore used plasma adiponectin as a surrogate drug marker for pioglitazone and aimed to achieve a similar adiponectin increase and treated mice with diets containing various pioglitazone doses (Supplementary Fig. 1). Based on these results, we selected a pioglitazone dose of 30 mg/kg/day (0.01875% in diet) for the preclinical study, which indeed caused an approximate 2-fold increase in plasma adiponectin levels compared to untreated mice (Fig. 2b).

### Tolvaptan, but not pioglitazone, improves renal survival and the cystic phenotype *in vivo*

**Figure 3:**
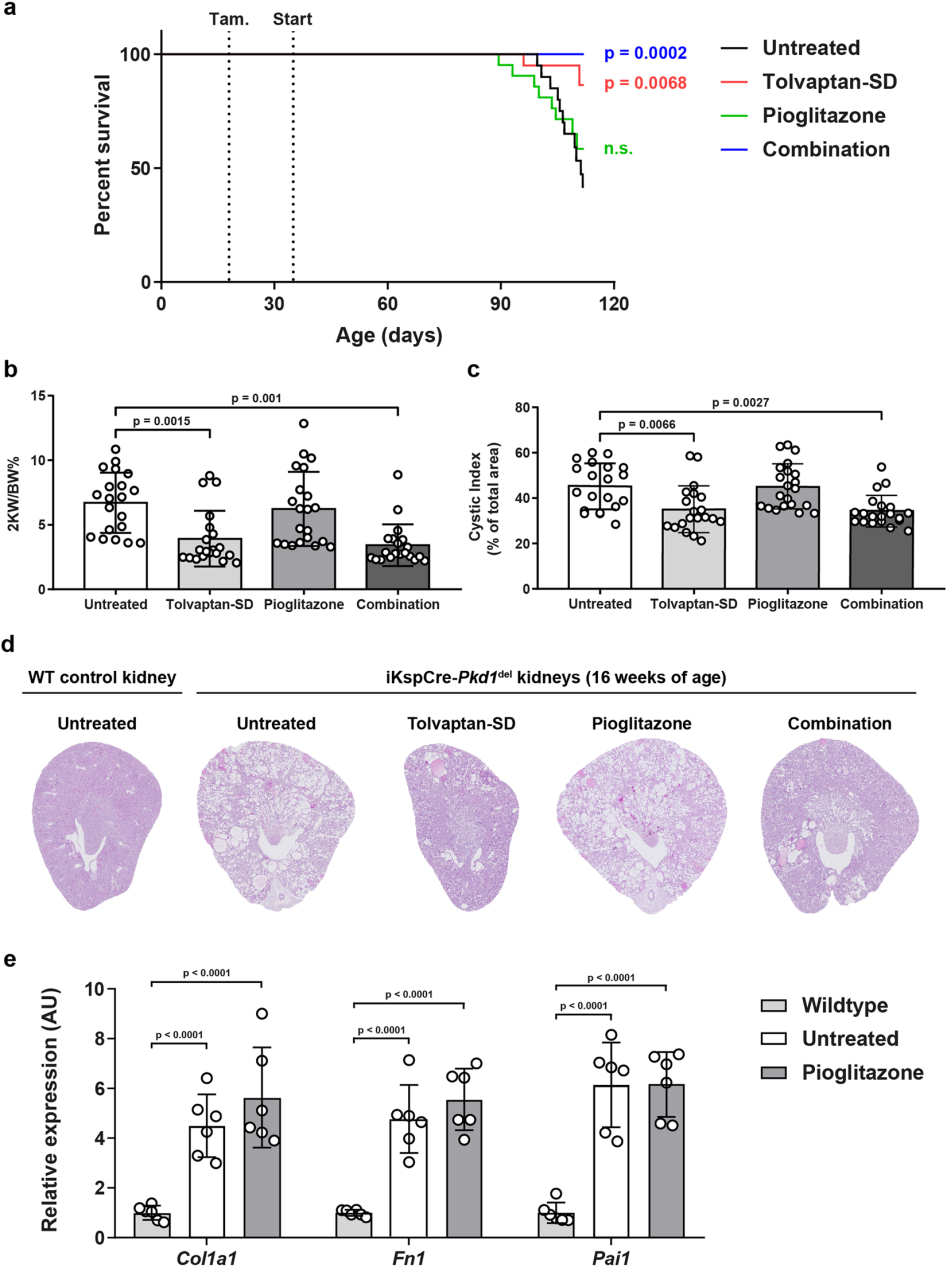
Tolvaptan improves renal survival and cystic parameters, while pioglitazone does not. **(a)** Renal survival of untreated mice (n = 20) versus treated mice (n = 20 or 21). **(b)** 2KW/BW% when mice reached ESRD or at the end of the experiment. **(c)** Cystic indices of mice at ESRD or at the end of the experiment. Differences between single-drug treatment with tolvaptan-SD and the combination treatment were non-significant. **(d)** Representative images of PAS-stained kidney sections from each treatment group. For each group, the image corresponding to the median 2KW/BW% is shown. **(e)** Gene expression of selected fibrosis markers in wildtype, untreated (i.e. cystic) iKspCre-*Pkd1*^del^ kidneys and pioglitazone-treated iKspCre-*Pkd1*^del^ kidneys. Each data point represents a single mouse. The Log Rank (Mantel-Cox) test was used for the Kaplan-Meier survival analyses. Data represent the mean ± SD. Significance was measured by one-way ANOVA followed by Tukey’s multiple comparisons test. n.s.: non-significant, 2KW/BW%: 2 kidneys weight to body weight ratio, WT: wildtype, AU: arbitrary units.

To investigate the effects of a combination treatment *in vivo*, we performed a preclinical study with our adult-onset PKD mouse model (iKspCre-*Pkd1*^del^, Fig. 2c)^43,44^. *Pkd1* gene deletion was induced at post-natal (PN) days 18 & 19 via administration of tamoxifen via oral gavage. Upon weaning, mice were randomly distributed over four experimental groups (i.e. untreated, tolvaptan-SD, pioglitazone and combination). Body weights of the mice did not differ between the different groups (Supplementary Fig. 2). The drug interventions started at 5 weeks of age using the selected doses as described above (i.e. 0.15% tolvaptan-SD and/or 0.01875% pioglitazone in the diet). From 14 weeks of age onward, we monitored renal function by weekly measurements of blood urea (BU) levels. Mice were sacrificed at the onset of end-stage renal disease (ESRD) (BU ≥ 20 mmol/L). Treatments continued until 50% of the untreated group had reached ESRD (50% ESRD), at which point the remaining mice from all groups were sacrificed. 50% ESRD in the untreated group was reached after 110 days of age (Supplementary Table 1). Single-drug treatment with tolvaptan-SD significantly improved renal survival compared to untreated mice (Fig. 3a). Moreover, the 2-kidney weight to body weight ratio (2KW/BW%), cystic indices and BU levels of tolvaptan-treated mice were all lower at the end of the experiment (Fig. 3b,c, Supplementary Fig. 2). Also, these mice showed an improved renal histology (Fig. 3d). Surprisingly, single-drug treatment with pioglitazone had no beneficial effect on any of these parameters and also failed to show any effect on the gene expression of multiple fibrotic markers (Fig. 3a–e, Supplementary Fig. 2). Although the combination treated mice tended to have a better renal survival rate compared to mice treated with tolvaptan, this difference was small and not statistically significant (p = 0.1325). Also, the other parameters (2KW/BW%, cystic index and BU levels) were all similar between the combination group and the tolvaptan group, indicating that the beneficial effect of the combination treatment was caused by tolvaptan alone. Therefore, despite an approximate 2-fold increase of plasma adiponectin (Fig. 2a), indicating successful administration of the drug in our experimental mice, pioglitazone did not have an effect on PKD progression.

### PPARγ signalling in the mouse kidney is unaffected by pioglitazone treatment

**Figure 4:**
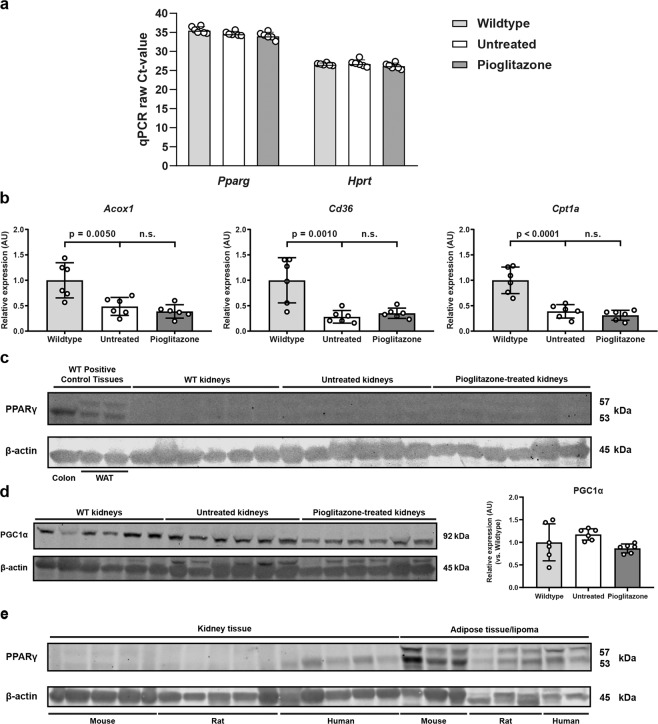
PPARγ signalling is not altered in cystic kidneys of pioglitazone-treated mice. **(a)** Raw Ct-values for the expression of *Pparg* and *Hprt* (internal housekeeping gene) in wildtype, untreated (i.e. cystic) iKspCre-*Pkd1*^del^ kidneys and pioglitazone-treated iKspCre-*Pkd1*^del^ kidneys. Higher Ct-values correspond with lower gene expression. Ct-values of 35 and higher are close to the detection limit. Each data point represents the average of three technical replicates of 1 mouse. **(b)** Gene expression of selected PPARγ target genes in kidneys of wildtype, untreated (i.e. cystic) iKspCre-*Pkd1*^del^ mice and pioglitazone-treated iKspCre-*Pkd1*^del^ mice. Expression of *Acox1*, *Cd36* and *Cpt1a* is significantly downregulated in untreated kidneys, when compared to wildtypes. This expression pattern is not corrected upon pioglitazone administration. *Hprt* expression was used as internal housekeeping gene. Data are shown as fold change compared to wildtype kidneys. Each data point represents the average of three technical replicates of 1 mouse. **(c)** Western blotting for PPARγ on protein extracts isolated from wildtype positive control tissues (colon and WAT), wildtype kidneys, untreated (i.e. cystic) iKspCre-*Pkd1*^del^ kidneys and pioglitazone-treated iKspCre-*Pkd1*^del^ kidneys. β-actin protein expression was used as an internal loading control. **(d)** Western blotting and quantification for PGC1α on protein extracts isolated from wildtype kidneys, untreated (i.e. cystic) iKspCre-*Pkd1*^del^ kidneys and pioglitazone-treated iKspCre-*Pkd1*^del^ kidneys. β-actin protein expression was used as an internal loading control. **(e)** Western blotting for PPARγ in protein extracts isolated from kidneys of wildtype mice, rats and human kidney samples, as well as from mouse and rat adipose tissue and human lipoma tissue as positive controls. β-actin protein expression was used as an internal loading control. No quantification of the blots presented in panels **(c)** and **(e)** is shown, as the signal intensity is too close to the background levels for an accurate quantification. Samples were run on the same gel, image acquisition for each protein was done separately. The blots presented in panels **(c**–**e)** are cropped, the original blots can be found in Supplementary Fig. 6. Data represent mean ± SD. Significance was measured by one-way ANOVA followed by Tukey’s multiple comparisons test. n.s.: non-significant, Ct: cycle threshold, AU: arbitrary units, WT: wildtype, WAT: white adipose tissue, kDa: kilodalton.

Next, we investigated the contradictory results of pioglitazone treatment between the 3D cyst assay and the mouse experiment. We hypothesized that the observed differences may be explained by differences in the expression level of the transcription factor PPARγ, an important and best described target of pioglitazone. At the mRNA level, *Pparg* expression in mouse kidneys is very low, compared to the expression of the housekeeping gene *Hprt* (Fig. 4a). At the protein level, PPARγ expression was easily detectable in colon tissue and white adipose tissue (WAT) in mice. However, PPARγ is barely expressed in mouse kidney homogenates (Fig. 4c). Next, we assessed whether pioglitazone treatment affected the expression of multiple downstream PPARγ targets in mouse kidneys. Mice with a similar 2KW/BW% were selected for this analysis to exclude any phenotypic bias. In line with previously published results^45^, the expression of various selected PPARγ target genes (*Acox1, Cd36 and Cpt1a)* were lower in cystic kidneys, compared to wildtype (WT) kidneys. Pioglitazone treatment was unable to restore their expression back to WT levels (Fig. 4b). Also, no effect of pioglitazone treatment was seen on the expression of peroxisome proliferator-activated receptor gamma coactivator-1 alpha (PGC1α), a master regulator of mitochondrial biogenesis, in mouse kidney homogenates (Fig. 4d). These results appear to suggest that pioglitazone failed to slow PKD due to low PPARγ expression in the kidney. However, although pioglitazone did inhibit cyst growth in 3D cyst cultures, in-house RNA-sequencing analysis surprisingly showed a complete lack of *Pparg* expression (counts per million (CPM) = 0), which was confirmed by qPCR (Supplementary Fig. 4). This indicates that PPARγ is not required for pioglitazone to reduce cyst swelling in 3D cysts.

### Pioglitazone target expression in mouse, rat and human kidneys

**Figure 5:**
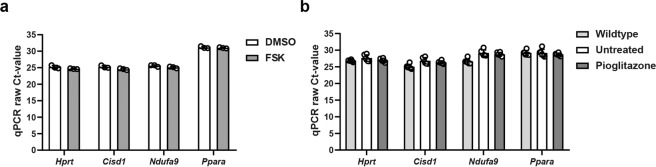
Gene expression of other pioglitazone targets in 3D cysts and in mouse kidneys. **(a)** Raw Ct-values for the expression of other pioglitazone targets (*Cisd1*, *Ndufa9*, *Ppara*) and *Hprt* (internal housekeeping gene) in DMSO- and FSK-treated 3D cysts. Higher Ct-values correspond with lower gene expression. Ct-values of 35 and higher are close to the detection limit. **(b)** Raw Ct-values for the expression of other pioglitazone targets (*Cisd1*, *Ndufa9*, *Ppara*) and *Hprt* (internal housekeeping gene) in wildtype, untreated (i.e. cystic) iKspCre-*Pkd1*^del^ kidneys and pioglitazone-treated iKspCre-*Pkd1*^del^ kidneys. Higher Ct-values correspond with lower gene expression. Ct-values of 35 and higher are close to the detection limit. Each data point represents the average of three technical replicates of 1 well **(a)** or 1 mouse **(b)**. Data represent mean ± SD. Ct: cycle threshold, FSK: forskolin.

Next, we aimed to further explore whether renal PPARγ expression could be predictive of pioglitazone efficacy. We analysed PPARγ expression in mouse, rat and human kidneys by consulting various publicly available microarray datasets^44,46,47^. All three species show very low *Pparg* expression, compared to the housekeeping gene *Hprt* (Supplementary Table 2). As comparing mRNA expression from different species and across different platforms is challenging, and may not be predictive for protein expression, we used Western blot analysis to compare PPARγ protein expression in mouse, rat and human kidney samples. As expected, PPARγ was easily detectable in white adipose tissue from all three species. Human kidney samples show a faint PPARγ expression, whereas in mouse and rat kidneys, PPARγ expression was barely detectable (Fig. 4e). Since PPARγ expression is very low in mouse and rat kidneys, it seems unlikely that pioglitazone efficacy in the PCK rat can be explained by PPARγ agonism in the kidney^38,39^. Aside from PPARγ, pioglitazone has also been reported to bind to the two mitochondrial proteins mitoNEET (encoded by *Cisd1*) and NADH dehydrogenase [ubiquinone] 1 alpha subcomplex subunit 9 (a subunit of complex I of the electron transport chain, encoded by *Ndufa9*), and to a lesser extent to PPARα^48–50^. Analysis of publicly available microarray data and qPCR analysis revealed that these targets are expressed abundantly in 3D-cultured cysts and kidneys from mouse, rat and human (Fig. 5a,b, Supplementary Table 2). These data might provide an explanation for pioglitazone efficacy in our 3D cyst assay and the PCK rat, but it does not explain the lack of efficacy in our mouse model. In addition, pioglitazone treatment did not alter the expression of any of these targets, suggesting that pioglitazone did not alter mitochondrial function in PKD kidneys (Supplementary Fig. 5). The question whether pioglitazone treatment can slow down disease progression in ADPKD patients therefore still remains.

## Discussion

Around 70% of ADPKD patients develop ESRD in their lifetime, making ADPKD the fourth most common renal disease requiring renal replacement therapy^51^. Tolvaptan is the only approved drug available for ADPKD patients at the moment. Unfortunately, the drug is only suited for specific patient groups. Therefore, it is critical to develop new therapeutic interventions which are both safe and effective.

Pioglitazone is a relatively safe drug that has been used for the treatment of type 2 diabetes and has shown efficacy in slowing PKD progression in the PCK rat. In this study, we therefore tested whether combining tolvaptan and pioglitazone in iKspCre-*Pkd1*^del^ mice could lead to enhanced efficacy on PKD progression. Although tolvaptan indeed slowed PKD, pioglitazone was surprisingly ineffective. This suggests that pioglitazone efficacy varies substantially between PKD models and species. There are a few possibilities that are crucial to understand this variability. First, the observed variability might be explained by potential differences in renal expression of pioglitazone targets. In the present study, we show that the expression of the most obvious pioglitazone target PPARγ across different models and species is not consistent with whether or not pioglitazone exerts cyst inhibiting effects. For example, pioglitazone slowed cyst growth in 3D-cultured cysts, but these cysts do not express *Pparg*. In addition, the expression of PPARγ in mouse and rat kidneys is comparably low and therefore does not explain why pioglitazone was effective in the PCK rat and not in mice. Other studies also provide evidence for fairly low renal *Pparg* expression in male Sprague-Dawley rat kidneys, with *Pparg* expression almost exclusively present in the collecting ducts of the inner medulla, a region known to be resistant to cyst growth^43,52^.

A different study showed that maternal administration of pioglitazone (80 mg/kg/day) inhibited cystogenesis in *Pkd1*^−/−^ embryonic kidneys^36^. The effective window of maternal pioglitazone administration was between E7.5 – E9.5. However, renal *Pparg* expression only starts after E14.5^52^, suggesting that either extra-renal effects (i.e. on the correction of cardiac defects and/or on subcutaneous oedema) or other pioglitazone targets explain the cyst inhibiting effects in the *Pkd1*^−/−^ embryos. Pioglitazone has also been reported to bind to mitoNEET (encoded by the *Cisd1* gene) with equally high affinity as PPARγ^49^. MitoNEET is a protein located in the outer mitochondrial membrane and is likely involved in mitochondrial bioenergetics and inflammation^53^. In addition, pioglitazone can bind to subunits of complex I of the mitochondrial respiratory chain (for example to *Ndufa9*)^50^. Pioglitazone has also been reported to stimulate PPARα activity, which has been shown to be involved in PKD progression^45,48,54^. However, although the PPARα agonist fenofibrate has been shown to slow cyst growth in PKD mouse models, the binding affinity of pioglitazone to PPARα is likely too low to sufficiently activate PPARα when used at clinically relevant doses^45,54^. In kidney samples of mice, rats and humans, and in 3D cyst cultures, mRNA expression of these pioglitazone targets appeared to be substantially higher than observed for PPARγ. Although these findings may at least explain some of the PPARγ-independent cyst-reducing effects of pioglitazone in our 3D cyst assay and in PCK rats, it does not provide an explanation for the lack of efficacy in our PKD mouse model.

A second possibility that may explain the variable efficacy of pioglitazone across species relates to the pharmacokinetics (PK) of pioglitazone. For example, pioglitazone is metabolized by the liver enzymes CYP2C8 and CYP3A4 and it has been shown that the expression of cytochrome P450 enzymes can differ between various organisms, including mice, rats and humans^55,56^. However, pioglitazone doses used in mouse, rat and human studies all lead to an approximate 2–3-fold upregulation of the surrogate drug marker adiponectin in the circulation, suggesting that the used doses lead to comparable circulating drug levels across the different species, with comparable effects on adipose tissue^31,32,57^.

Also, other pharmacodynamic (PD) parameters cannot be ruled out, this may include how the drug is taken up by the kidney, potential renal signalling differences between PKD models that affect the response to pioglitazone, or extra-renal effects that may impact how pioglitazone affects PKD progression in the kidney. For example, one obvious difference between the mice used in this study and the PCK rat, is that in our mice, the *Pkd1* gene, associated with ADPKD, is disrupted, whereas in the PCK rat, the *Pkhd1* gene, associated with ARPKD, is mutated. However, although our PKD mice and PCK rats seem to respond differently to pioglitazone, several other data do suggest an overlap in important PKD related mechanisms. Inflammatory processes, increased proliferation and altered fatty acid metabolism are all shared between PCK rats and our PKD mice^44,47^. At the molecular level, mTOR and cAMP related signalling has been shown to drive cyst growth in many models of PKD and both the PCK rat and our PKD mice are capable to respond to inhibitors of those signalling pathways (i.e. sirolimus as an mTOR inhibitor and tolvaptan as a vasopressin V2 receptor antagonist capable of reducing [cAMP]i)^7,58^. Although we cannot rule out that subtle mechanistic differences between our mice and the PCK rat affect pioglitazone efficacy, the available data do suggest that cyst growth in both models are driven by common pathways.

In addition, extra-renal effects of pioglitazone may indirectly affect cyst progression and these extra-renal effects may differ across species. Extra-renal effects of pioglitazone that can be conceivably linked to mechanisms associated with PKD progression include its anti-hypertensive effects, and its known ability to improve glycaemic and lipid profiles of particularly type 2 diabetes patients^36,37,59,60,61–66^. However, whether such extra-renal effects elicited by pioglitazone indeed have the potential to significantly slow PKD progression in some PKD models is not clear and will require additional studies.

Collectively, our data show that tolvaptan-SD slowed cyst progression in an adult-onset iKspCre-*Pkd1*^del^ mouse model as expected. However, pioglitazone administration at a dose that led to plasma adiponectin levels similar to those of studies using rats or humans, did not slow PKD in iKspCre-*Pkd1*^del^ mice. The discordant results between several PKD rat studies and our study could not be explained by renal expression patterns of known pioglitazone targets, which were similar across various species. However, we cannot rule out that several of the discussed PK/PD parameters may differ between species and determine whether or not pioglitazone is capable to slow PKD progression. The ongoing clinical trial with low-dose pioglitazone treatment (ClinicalTrials.gov number, NCT02697617) may provide more clarity whether pioglitazone is able to slow PKD progression in ADPKD patients.

## Material and Methods

### 3D cyst culture

3D cyst cell culture and drug treatments were performed as described before^41^, with minor optimizations. In short, mIMCD3-*Pkd1*^−/−^ (mIMRFNPKD 5E4) cells were expanded in cell culture medium (DMEM/F12 (Ham’s) culture medium (D8062, Sigma-Aldrich, Zwijndrecht, the Netherlands), supplemented with 10% fetal bovine serum (FBS, F7524, Sigma-Aldrich), GlutaMAX™ (35050038, Gibco, Life Technologies, Bleiswijk, the Netherlands), and penicillin/streptomycin (15140122, Gibco, Life Technologies)) for 72 hours. Cells were then washed with 1x phosphate-buffered saline (PBS), trypsinized with 1x trypsin-EDTA (T4174, Sigma-Aldrich) and mixed with Cyst-Gel (OcellO B.V., Leiden, the Netherlands) to a final concentration of 150000 cells/mL. The cell-gel mixture was then pipetted to 384-well plates (Greiner Clear, Greiner Bio-One B.V., Alphen aan den Rijn, the Netherlands) using Cybi Felix robotic liquid handler (Analytic Jena AG). After polymerization of the gel, cell culture medium was added. Cells were allowed to form cysts for 96 hours, after which cysts were exposed to vehicle (0.2% DMSO), 2.5 μM forskolin (34427, Calbiochem, Millipore B.V., Amsterdam, the Netherlands) and drug treatments for 72 hours. Afterwards, cysts were fixed with 4% formaldehyde (Sigma-Aldrich) and simultaneously permeabilized with 0.2% Triton-X100 (Sigma-Aldrich) and stained with 0.25 M rhodamine-phalloidin (Sigma-Aldrich) and 0.1% Hoechst 33258 (Sigma-Aldrich) in 1x PBS for 12 hours at 4 °C in the dark. Imaging was done using the Molecular Devices ImageXpress Micro XLS (Molecular Devices) with a 4x NIKON objective. For both channels, between 30–35 images throughout the entire z-stack were made for each well, 50 µm apart. Each individual image was analysed using Ominer™ image analysis software (OcellO B.V.) integrated in KNIME Analytics platform (Konstanz, Germany, http://www.knime.org/). The automated calculation of various phenotypic characteristics related to cyst area and cytotoxicity was performed as described before^41^.

### Mouse experimental procedures

Tamoxifen-inducible kidney-specific *Pkd1* deletion (iKspCre-*Pkd1*^del^) mice (tam-*KspCad-CreER*^*T2*^; *Pkd1*^*del2-1l/lox2-11*^) were generated in-house as described before^67^. Kidney-specific deletion of the *Pkd1* gene was induced via oral gavage of 150 mg/kg tamoxifen (T5648, Sigma-Aldrich, dissolved in absolute ethanol) dissolved in sunflower oil on PN18 and 19^44^. Upon weaning (PN27–28), male mice treated with tamoxifen were randomized by body weight over 4 groups: untreated (n = 20), tolvaptan-SD (n = 20), pioglitazone (n = 21) or combination of tolvaptan-SD and pioglitazone (n = 20). Only male mice were used for the experiment, as they display a more severe disease progression. The number of animals per group was calculated with a power calculation, based on previously acquired data with this model. Tolvaptan-SD is a tolvaptan formulation designed to improve the drug’s oral bioavailability (information kindly provided by Otsuka Pharmaceuticals, Tokyo, Japan). Interventions started at 5 weeks of age (PN35), with the untreated group receiving manually prepared food pellets produced from a powdered diet (RM3 (E) FG, Special Diet Services, Witham, United Kingdom). Treatment groups received similarly prepared food pellets, supplemented with either 0.15% tolvaptan-SD, which is the equivalent of the commonly used dose in preclinical research of 0.1% tolvaptan (information provided by Otsuka Pharmaceuticals) and/or 0.01875% pioglitazone (I868, AK Scientific, Union City, CA, USA, resulting in a dose of 30 mg/kg/day based on an average food intake of 4 grams per mouse per day). The diuretic effect of tolvaptan was evaluated by measuring the water intake (by weighing the water bottles) in the first two weeks after treatment started. Water intake per mouse per day was calculated by dividing the difference in water bottle weight by the number of days between measurements and the number of mice per cage. Mice were housed in groups of 2 or 3 animals per cage, with *ad libitum* access to food and water. Housing and husbandry details are provided in Supplementary Table 3. Kidney function was monitored from 14 weeks of age onward via weekly blood urea measurements using the Reflotron^®^ Sprint (Roche Diagnostics, Mannheim, Germany). Mice were considered to reach ESRD at a BU level of 20 mmol/L (calculated by linear interpolation between the last two measurements). Mice were then sacrificed and tissues of interest were removed and snap-frozen in liquid nitrogen. Kidneys were first weighed for calculation of 2KW/BW%, bisected and either snap-frozen in liquid nitrogen or fixed in 4% buffered formaldehyde. Snap-frozen tissues were stored at −80 °C until further use. All the animal experiments were approved by the Animal Experiment Ethics Committee of Leiden University Medical Center and the Commission Biotechnology in Animals of the Dutch Ministry of Agriculture. All methods were performed in accordance with their guidelines and regulations.

### Rat experimental procedures

Male Wistar rats (surplus to experimental use) were obtained via the Leiden University Medical Center animal facility. Housing and husbandry details are provided in Supplementary Table 3. Rats were sacrificed and tissues of interest were removed and snap-frozen in liquid nitrogen. Kidneys were first bisected before being snap-frozen in liquid nitrogen. Snap-frozen tissues were stored at −80 °C until further use. All the animal experiments were approved by the Animal Experiment Ethics Committee of Leiden University Medical Center and the Commission Biotechnology in Animals of the Dutch Ministry of Agriculture. All methods were performed in accordance with their guidelines and regulations.

### Human tissue sample acquisition

Human kidney tissue samples were obtained from donor kidneys non-suitable for transplant. Human lipoma samples were obtained from the remaining tumour material left after samples were taken for diagnostics. Informed consent was obtained from all participants and/or their legal guardian/s. All tissue samples were obtained and handled in accordance with institutional guidelines approved by the Commission Medical Ethics of Leiden University Medical Center (institutional review board) and with the Code of Conduct regarding the responsible use of human tissues.

### Plasma adiponectin measurements

When mice reached ESRD or at the end of the experiment, blood samples were drawn in heparin-coated capillaries (Microvette^®^ CB 300 LH, Sarstedt, Nümbrecht, Germany) and subsequently centrifuged for 5 minutes at 8000 × g. Plasma was collected and stored at −80 °C until further use. Diluted plasma was assayed for adiponectin levels using a commercially available ELISA kit (MRP300, R&D Systems, Abingdon, United Kingdom) according to manufacturer’s instructions.

### Histology

Tissues fixed overnight in 4% buffered formaldehyde were embedded in paraffin. Sections were stained with Periodic acid-Schiff (PAS) using standard protocols. Cystic indices were calculated as previously described^9,44,68^. In short, a specific colour pallet is designed with Photoshop software (Adobe Systems Inc., San Jose, CA, USA) to remove all pixels in the cyst lumen, leaving only the pixels of the kidney tissue. The cystic index was then calculated by the percentage of ‘cyst lumen’ pixels versus the total amount of pixels in the image.

### RNA isolation and quantitative PCR

Snap-frozen tissues were homogenised using a MagNA Lyser instrument (Roche Life Science, Basel, Switzerland) in MagNA Lyser Green Beads tubes (Roche Life Science), followed by total RNA isolation from the kidney homogenate using Tri-Reagent (Sigma-Aldrich). Total RNA was reverse transcribed to cDNA with the Transcriptor First Strand cDNA Synthesis Kit (Roche Life Science), and qPCR was performed using the FastStart Universal SYBR Green Master (Rox) (Sigma-Aldrich), according to the manufacturer’s protocol. mRNA expression was normalized to *Hprt* expression and expressed as a fold change using the ΔΔCT method. The primer sequences used are listed in Supplementary Table 4.

### Protein isolation and western blotting

Snap-frozen tissues were homogenised in RIPA buffer (50 mM Tris-HCl pH 7.4, 150 mM NaCl, 1 mM EDTA) supplemented with protease inhibitor cocktail (#05892970001, Roche Diagnostics) using MagNA Lyser technology described above. Sample buffer supplemented with β-mercaptoethanol was then added for protein denaturation in the following final concentrations: 100 mM Tris-HCl pH 7.4, 4% SDS, 12% glycerol, 0.008% bromophenol blue and 4% β-mercaptoethanol. Lysates were stored at −80 °C until further use. Proteins were separated via SDS-PAGE, followed by transfer to a nitrocellulose membrane (Bio-Rad Laboratories, Veenendaal, The Netherlands). Membranes were blocked for 1 hour at room temperature in 25% SEA BLOCK Blocking Buffer (37527, ThermoFisher Scientific, Rockford, IL, United States) in Tris-buffered saline (TBS), followed by overnight incubation at 4 °C with primary antibodies against PPARγ (2435, Cell Signaling, Leiden, the Netherlands), PGC1α (ab54481, Abcam, Cambridge, United Kingdom) or β-actin (4967, Cell Signaling). Blots were then washed with 0.1% Tween-20 in TBS (TBS-T) and incubated for 1 hour at room temperature with goat-anti-rabbit IRDye 800CW (926–32211, LI-COR Biosciences, Bad Homburg, Germany) secondary antibody. Blots were visualized and scanned with the Odyssey CLx Imaging System (Li-COR Biosciences). Protein content was quantified via densitometric analysis (Image Studio Lite, Li-COR Biosciences), normalized to β-actin protein content and expressed as a fold change.

### Statistical analysis

Statistical analyses were performed with GraphPad Prism 8 (GraphPad Software, La Jolla California USA, www.graphpad.com). All results are expressed as mean ± SD, unless stated otherwise in the figure legends. The Log Rank (Mantel-Cox) test was used for the Kaplan-Meier survival analyses. Comparisons between 2 groups were done using the two-tailed unpaired Student’s *t*-test, while comparisons between multiple groups were done using the one-way ANOVA, followed by Tukey’s or Dunnett’s multiple comparison test. Results were considered statistically significant when P < 0.05.

## Supplementary information

Supplementary Figures.

Supplementary Tables.

## Data Availability

The microarray datasets analysed in the current study are available in the Gene Expression Omnibus repository (GEO, http://www.ncbi.nlm.nih.gov/geo/, IDs: GSE126454; GSE33056 and GSE7869). All data generated or analysed during this study are included in this published article (and its Supplementary Information files).
