# Supplementary material for: Renal cyst growth is attenuated by a combination treatment of tolvaptan and pioglitazone, while pioglitazone treatment alone is not effective
Source: Sci Rep. 2020 Feb 3;10:1672. doi: 10.1038/s41598-020-58382-z (PMC6997373; doi:10.1038/s41598-020-58382-z)
Supplement: Supplementary file 1 — Supplementary Figures. [file 41598_2020_58382_MOESM1_ESM.pdf]

Renal cyst growth is attenuated by a combination treatment of tolvaptan and pioglitazone, while pioglitazone treatment alone is not effective

Anish A. Kanhai[1], Hester Bange[2], Lotte Verburg[1,3], Kyra L. Dijkstra[1,3], Leo S. Price[2], Dorien J. M. Peters[1*] and Wouter N. Leonhard[1]

[1]Department of Human Genetics, Leiden University Medical Center, Leiden, the Netherlands
[2]OcellO B.V., Leiden, the Netherlands
[3]Department of Pathology, Leiden University Medical Center, Leiden, the Netherlands

*Corresponding author: Dorien J.M. Peters, Department of Human Genetics, Leiden University Medical Center, Leiden, the Netherlands. d.j.m.peters@lumc.nl

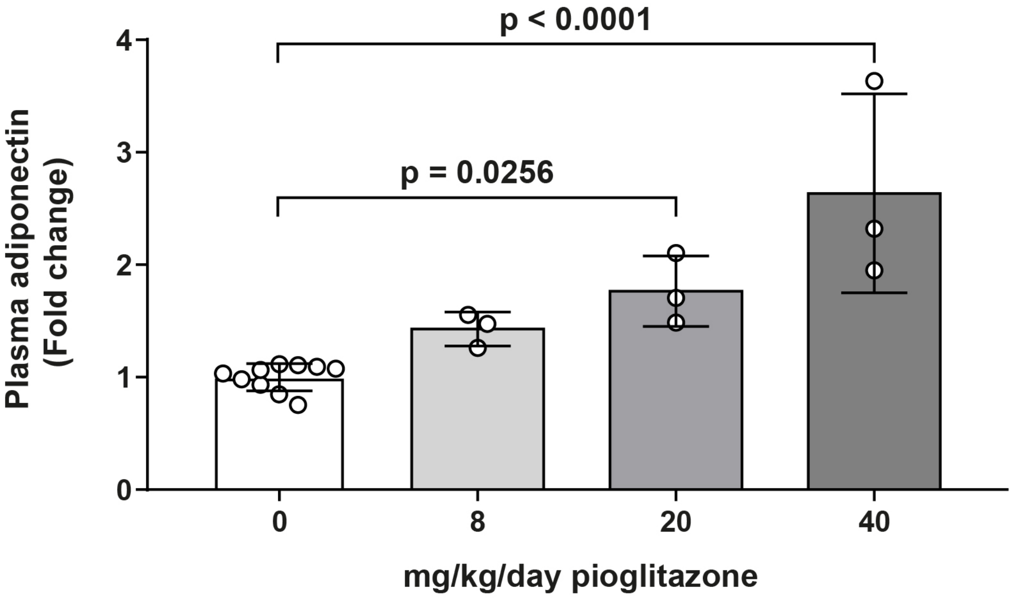

Supplementary Figure 1: Pioglitazone dose-dependently increases plasma adiponectin concentrations.
Wildtype mice were administered with diets containing increasing doses of pioglitazone. Plasma adiponectin concentrations were measured, with each data point representing the average plasma concentration of 1 mouse, measured in duplicate. Data are shown as fold change compared to the untreated group (0 mg/kg/day pioglitazone). Data represent the mean ± SD. Significance was measured by one-way ANOVA followed by Tukey's multiple comparisons test.

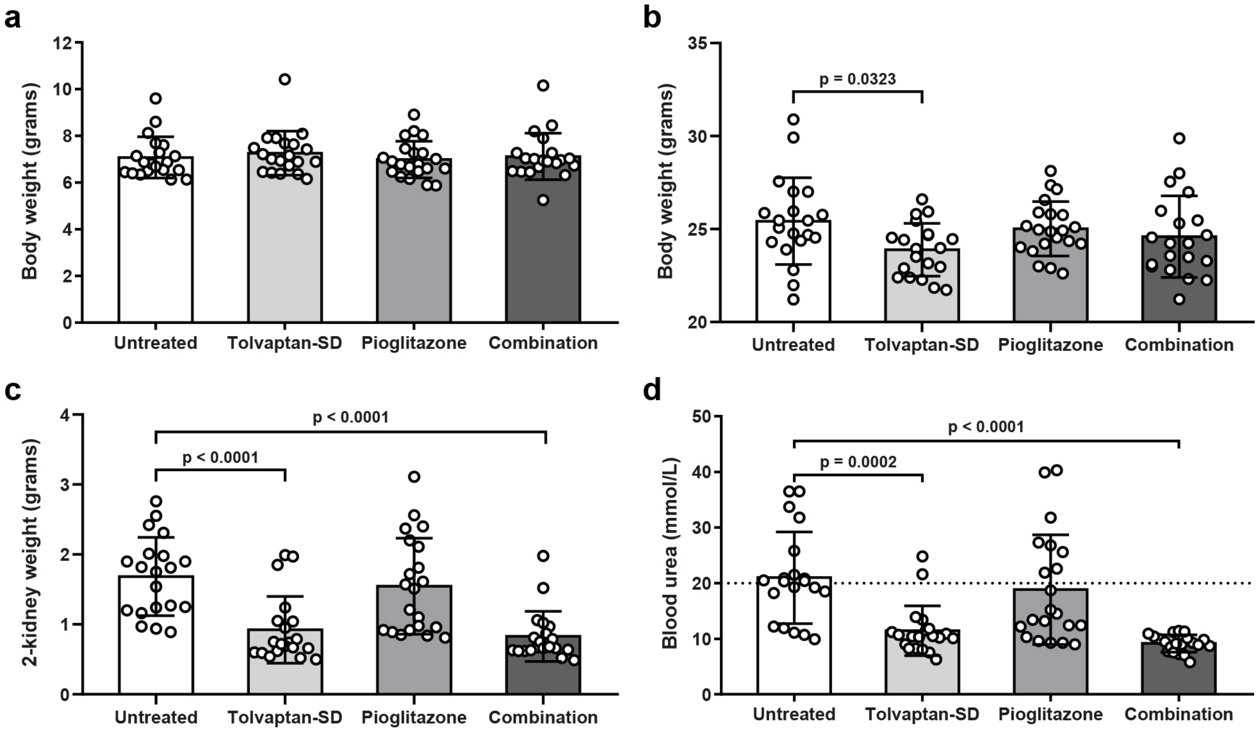

Supplementary Figure 2: Additional mouse data from preclinical study
(a) Body weight of all mice at the start of the preclinical study after randomization. The body weight of all groups did not significantly differ from each other. **(b)** Body weight of all mice at their respective endpoints. **(c)** 2-kidney weight of all mice at their respective endpoints. **(d)** Blood urea concentrations (mmol/L) of all mice at their respective endpoints. Each data point represents a single mouse. The difference between single-drug treatment with tolvaptan-SD and the combination treatment was non-significant. Data represent the mean ± SD. Significance was measured by one-way ANOVA followed by Tukey's multiple comparisons test.

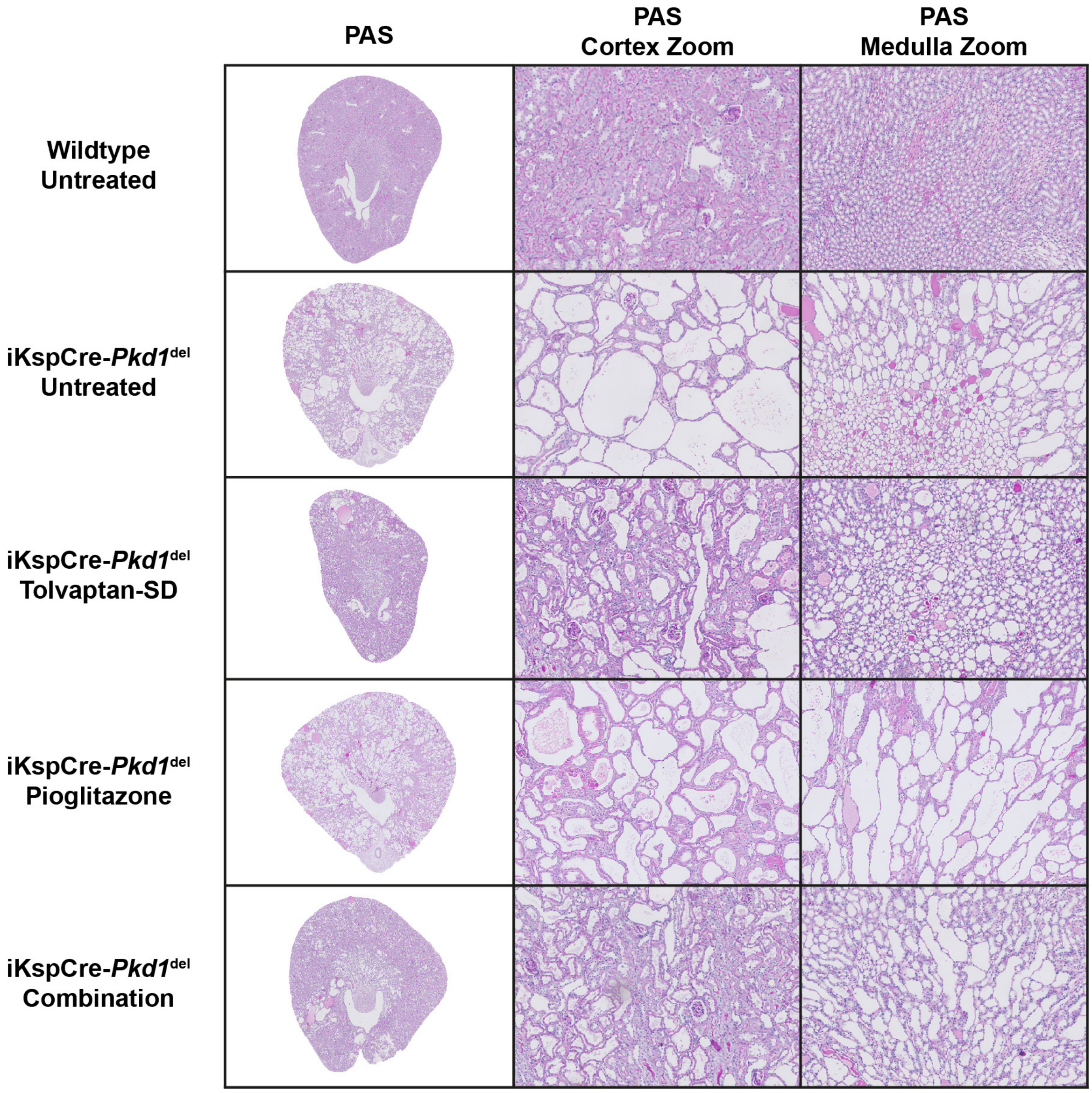

Supplementary Figure 3: Higher magnification images of histology images
Enlarged images of the PAS-stained kidney section from each treatment group (shown in Figure 3D) are shown. For each kidney, a magnified image of the cortex and medulla region is shown.

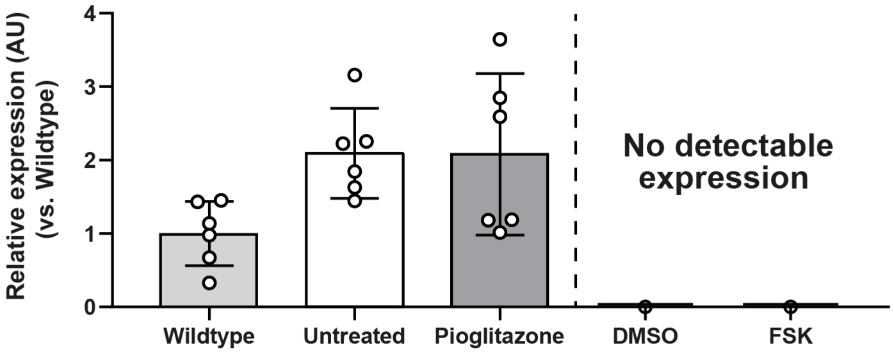

Supplementary Figure 4: Gene expression of *Pparg* in mouse kidneys (left) and 3D-cultured mIMCD3-*Pkd1*$^{-/-}$ cells (right)

Expression of *Pparg* in kidneys of wildtype, untreated (i.e. cystic) iKspCre-*Pkd1*^{del} mice, pioglitazone-treated iKspCre-*Pkd1*^{del} mice, DMSO- and FSK-treated 3D cysts. In mouse kidneys, *Pparg* expression was detectable in all groups, while in parallel in 3D cysts, no expression of the *Pparg* gene was detectable via qPCR. *Hprt* expression was used as internal housekeeping gene. Data are shown as fold change compared to wildtype kidneys. Data represent the mean ± SD. Significance was measured by one-way ANOVA followed by Tukey's multiple comparisons test. AU: arbitrary units, FSK: forskolin.

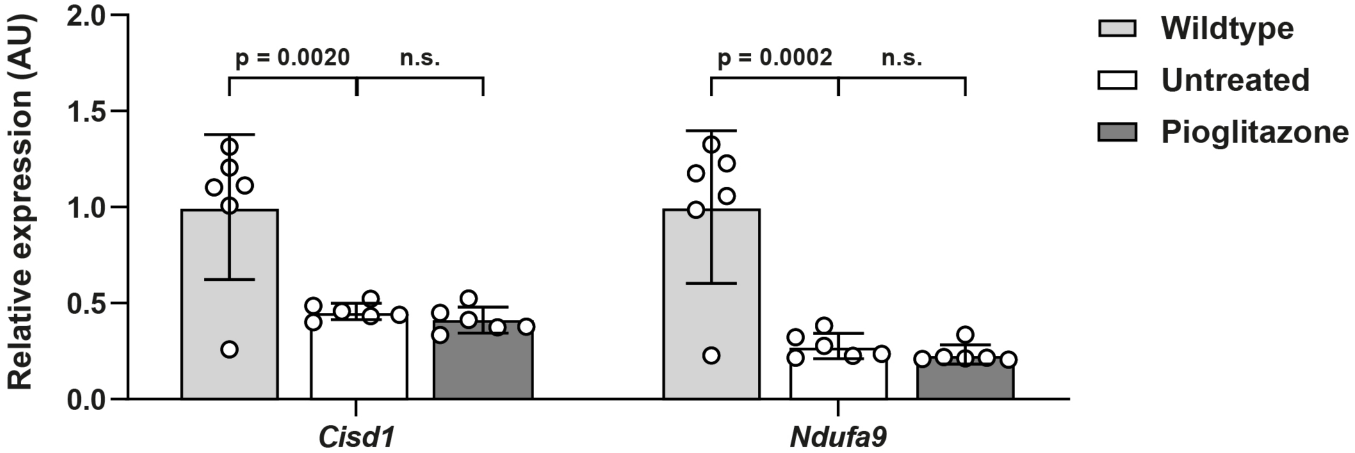

Supplementary Figure 5: Gene expression of other pioglitazone targets

Expression of genes encoding pioglitazone protein targets in kidneys of wildtype, untreated (i.e. cystic) iKspCre-*Pkd1*^{del} mice and pioglitazone-treated iKspCre-*Pkd1*^{del} mice. Expression of *Cisd1* and *Ndufa9* is significantly downregulated in untreated kidneys, when compared to wildtypes. This expression pattern is not corrected upon pioglitazone administration. *Hprt* expression was used as internal housekeeping gene. Data are shown as fold change compared to wildtype kidneys. Data represent the mean ± SD. Significance was measured by one-way ANOVA followed by Tukey's multiple comparisons test. AU: arbitrary units.

Figure 4C

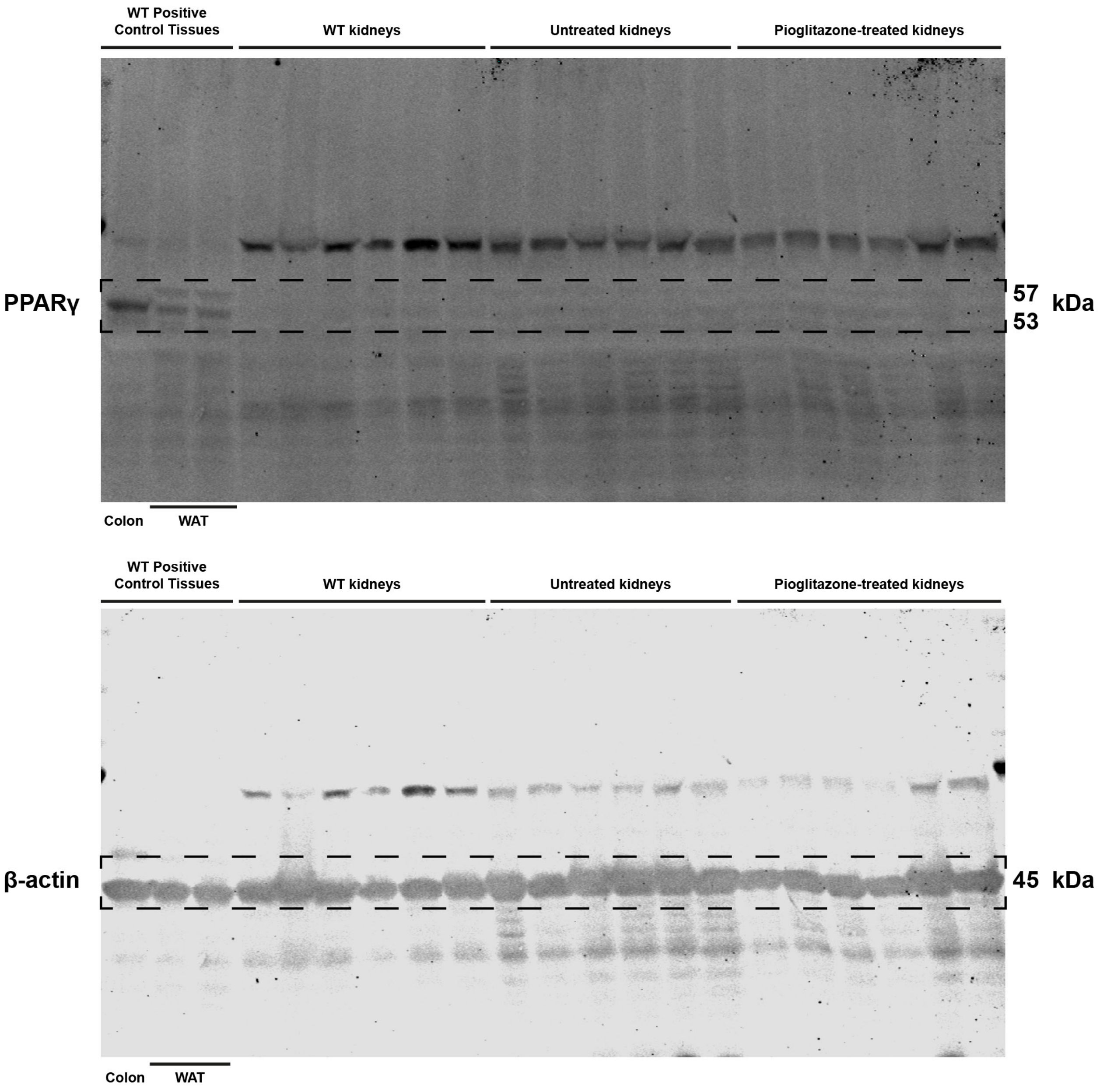

WT Positive Control Tissues
WT kidneys
Untreated kidneys
Pioglitazone-treated kidneys
PPARγ
57
53
kDa
Colon
WAT
WT Positive Control Tissues
WT kidneys
Untreated kidneys
Pioglitazone-treated kidneys
β-actin
45 kDa
Colon
WAT

Figure 4D

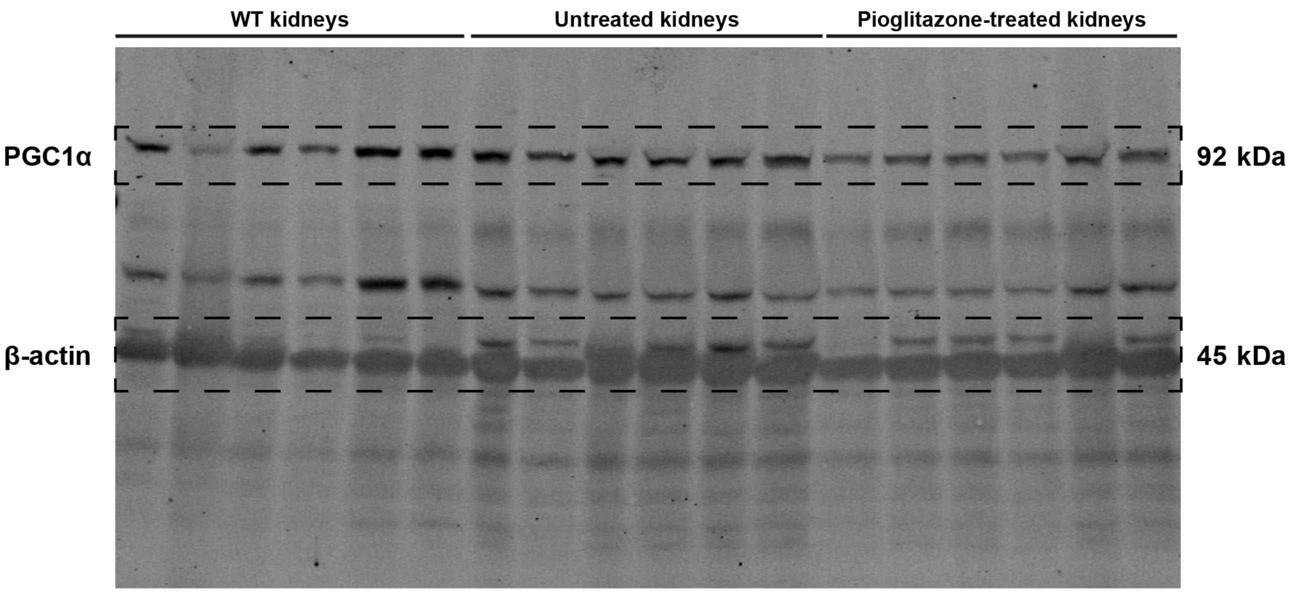

Figure 4E

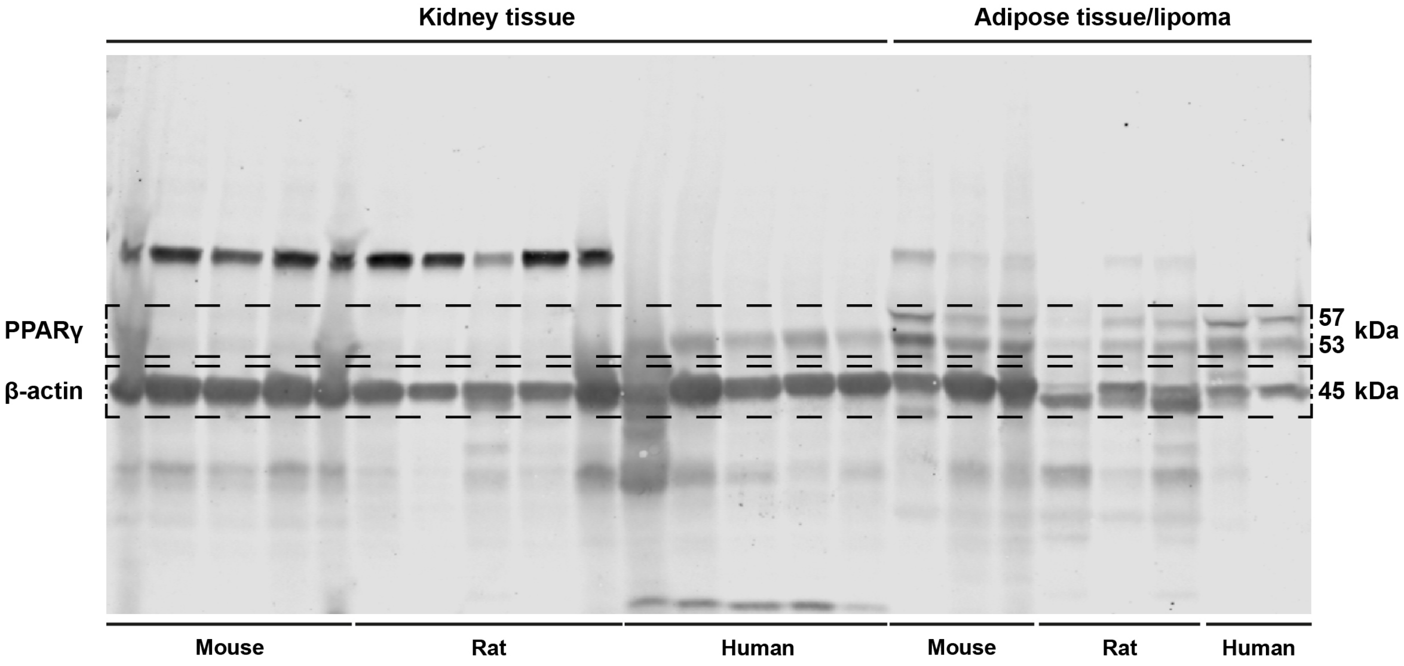

Supplementary Figure 6: Full size Western blots of the cropped blots presented in Figures 4C, 4D and 4E.

The dotted lines indicate the band of interest shown in the main figure. Samples were run on the same gel, image acquisition for each protein was done separately.
