# Supplementary material for: Renal cyst growth is attenuated by a combination treatment of tolvaptan and pioglitazone, while pioglitazone treatment alone is not effective
Source: Sci Rep. 2020 Feb 3;10:1672. doi: 10.1038/s41598-020-58382-z (PMC6997373; doi:10.1038/s41598-020-58382-z)
Supplement: Supplementary file 2 — Supplementary Tables. [file 41598_2020_58382_MOESM2_ESM.docx]

**Renal cyst growth is attenuated by a combination treatment of tolvaptan and pioglitazone, while pioglitazone treatment alone is not effective**

Anish A. Kanhai^1^, Hester Bange^2^, Lotte Verburg^1,3^, Kyra L. Dijkstra^1,3^, Leo S. Price^2^, Dorien J. M. Peters^1*^ and Wouter N. Leonhard^1^

^1^ Department of Human Genetics, Leiden University Medical Center, Leiden, the Netherlands

^2^ OcellO B.V., Leiden, the Netherlands

^3^ Department of Pathology, Leiden University Medical Center, Leiden, the Netherlands

*Corresponding author: Dorien J.M. Peters, Department of Human Genetics, Leiden University Medical Center, Leiden, the Netherlands. [d.j.m.peters@lumc.nl](mailto:d.j.m.peters@lumc.nl)

**Supplementary Table 1: Mice that reached ESRD per treatment group.**

Percentages of mice that reached ESRD at a specific timepoint in the preclinical study. In brackets, the number of mice with ESRD over the total number of mice per treatment group is shown.

|  | **Age (80 days)** | **Age (90 days)** | **Age (100 days)** | **Age (110 days)** |
| --- | --- | --- | --- | --- |
| **Untreated** | 0% (0/20) | 0% (0/20) | 5.0% (1/20) | 55.0% (11/20) |
| **Tolvaptan-SD** | 0% (0/20) | 0% (0/20) | 5.0% (1/20) | 10.0% (2/20) |
| **Pioglitazone** | 0% (0/21) | 4.8% (1/21) | 14.3% (3/21) | 38.1% (8/21) |
| **Combination** | 0% (0/20) | 0% (0/20) | 0% (0/20) | 0% (0/20) |

**Supplementary Table 2: Gene expression of pioglitazone targets across species**

Publicly available microarray datasets (see main text for references) were analysed to compare possible differences in gene expression of pioglitazone targets between species. Reported log2 values were converted to absolute values, and then normalized to the expression of the internal housekeeping gene *Hprt*.

| **Model** | ***Pparg/Hprt*** | ***Cisd1/Hprt*** | ***Ndufa9/Hprt*** | ***Ppara/Hprt*** |
| --- | --- | --- | --- | --- |
| iKspCre-*Pkd1*^del^  wildtype mice (n = 7) | 0,046 | 0,266 | 0,865 | 0,206 |
| iKspCre-*Pkd1*^del^ knockout mice (n = 12) | 0,043 | 0,171 | 0,536 | 0,114 |
| Sprague-Dawley  rats (n = 6) | 0,061 | 29,096 | 7,118 | 0,080 |
| PCK rats (n = 6) | 0,063 | 13,174 | 3,550 | 0,029 |
| Renal cortical tissue samples (n = 3) | 0,050 | 0,678 | 0,830 | 0,228 |
| Minimally cystic renal tissue  samples (n = 5) | 0,053 | 0,953 | 0,953 | 0,264 |
| Cystic renal tissue samples (n = 13) | 0,053 | 1,320 | 1,223 | 0,475 |

**Supplementary Table 3: Housing and husbandry details for animal experiments**

| \| **Housing details** \| \| \| --- \| --- \| \| **Type of facility** \| The central animal facility of the LUMC is a multi-barrier facility consisting of six units, including a barrier breeding unit, a transgenic facility and experimental units at biosafety level D1, DMII and DMIII. The Central Animal Facility should be considered as an SPF facility. The 'FELASA recommendations for the health monitoring of mouse, rat, hamster, guinea pig and rabbit colonies in breeding and experimental units' (M. Mähler, M. Berard et al., Lab. Anim. 48(3):178-192, 2014)' is considered as an exclusion list. \| \| **Type of cage/housing (mice)** \| Sealsafe® PLUS, green line system (Tecniplast S.p.A.). GM500 cage. \| \| **Type of cage/housing (rats)** \| Sealsafe® IVC, blue line system (Tecniplast S.p.A.), 1291H and 1500U. \| \| **Air ventilation unit/IVC system** \| + pressured; 75 ACH (Air changes/hour)'  - pressure; 75 ACH (Air changes/hour) in case of housing animals exposed to toxic compounds \| \| **Bedding material** \| LIGNOCEL® BK 8-15 (BK-8-15-00433, JRS) Only autoclaved in case of housing rats \| \| **Husbandry details** \| \| \| **Breeding programme** \| Standard nucleus breeding consists of at least 3 independent breeding pairs. Each pair is allowed to deliver maximum of four litters. Ideally, animals born from the 3rd litter are used to replace the existing breeding couples. \| \| **Breeding expansion programme** \| Production breeding pairs are kept in breeding until the female has given birth to a maximum of 6 litters (adjusted according to strain specific phenotypes). Litters will be used for expansion or for experiments. \| \| **Light/dark cycle** \| 06:30 – 07:00h sunrise, 07:00 – 18:00h daytime, 18:00 – 18:30h sunset.  Light intensity measured on top of the IVC racks: 40-80 LUX and 10-30 LUX, measured inside bottom and top cage \| \| **Air ventilation rate in holding rooms** \| 15 \| \| **Animal holding room temperature** \| 20 °C – 22 °C \| \| **Quality of water** \| Bottled tap water, provided in plastic drinking bottles (Tecniplast S.p.A.). Drinking bottles are refreshed weekly. \| \| **Type of food** \| RM3 diet, pelleted, irradiated 9kGy (801203, SDS diets). Treatment groups received similarly prepared food pellets supplemented with drugs (see Material & Methods) \| \| **Access to food and water** \| Ad libitum \| \| **Environmental enrichment** \| - Bed-r’Nest® (BRN8SR, The Andersons), irradiated 15kGy - GLP fun tunnels (mini 1022006, LBS Biotech). - Gnawing sticks (NGS E-021, Abedd). \| | | | | | | | |
| --- | --- | --- | --- | --- | --- | --- | --- | --- | --- | --- | --- | --- | --- | --- | --- | --- | --- | --- | --- | --- | --- | --- | --- | --- | --- | --- | --- | --- | --- | --- | --- | --- | --- | --- | --- | --- | --- | --- | --- |
|  |  |  |  |  |  |  |  |
|  | | | | | | | |
|  | | | | | | | |

**Supplementary Table 4: Primer sequences used for described qPCR experiments**

| **Gene name** | **Forward primer sequence** | **Reverse primer sequence** |
| --- | --- | --- |
| ***Acox1*** | CCGCCACCTTCAATCCAGAG | CAAGTTCTCGATTTCTCGACGG |
| ***Cd36*** | ATGGGCTGTGATCGGAACTG | TTTGCCACGTCATCTGGGTTT |
| ***Cisd1*** | TAGAATGGCACCCAACCCAC | GGCCGATGCCATGGATATGA |
| ***Col1a1*** | TGACTGGAAGAGCGGAGAGT | AGACGGCTGAGTAGGGAACA |
| ***Cpt1a*** | CACCAGTGATGATGCCATTCT | CTCCGCCTGAGCCATGAAG |
| ***Fn1*** | AATCCAGTCCACAGCCATTCC | CCTGTCTTCTCTTTCGGGTTCA |
| ***Hprt*** | ggctataagttctttgctgacctg | aacttttatgtcccccgttga |
| ***Ndufa9*** | GGACAAGGTGGAGCGGATAC | GACAGCCATCGGTAGGTACG |
| ***Pai1*** | GCCAACAAGAGCCAATCAC | ACCCTTTCCCAGAGACCAG |
| ***Ppara*** | CCTCAAAGTCTGAGCGGTCT | CTAACCTTGGGCCACACCT |
| ***Pparg*** | GACCGAGTGTGACGACAAGATT | AGAGCTGATTCCGAAGTTGGTG |
